# An approach to evaluating scrotal skin-based lesions: A case report of Basal Cell Carcinoma of the Scrotum in patient with multiple risk factors

**DOI:** 10.1016/j.eucr.2022.102130

**Published:** 2022-06-17

**Authors:** Jessica Teoh, Alexander Combes, Brayden March, Geoff Watson, Paul Sved

**Affiliations:** a Department of Urology, Royal Prince Alfred Hospital, Camperdown, NSW, 2050, Australia; b Department of Pathology, Royal Prince Alfred Hospital, Camperdown, NSW, 2050, Australia

## Abstract

Basal cell carcinoma (BCC) is rare on non-sun exposed skin such as the scrotum and thus diagnosis is often delayed. This case highlights an approach to scrotal skin lesions, risk factors and diagnostic features of BCC. Importantly, scrotal BCCs are more likely to metastasise than non-scrotal BCCs. Management should consist of wide local excision and recommended follow up with thorough clinical history, skin examination and imaging in high-risk patients.

## 1 Introduction

Basal cell carcinoma accounts for 80% of non-melanoma skin cancers however less than 1% are located on non-sun exposed skin of the genitalia.^1^ Approximately 100 cases of scrotal BCCs have been reported to date and importantly demonstrate higher propensity for metastasis than non-scrotal BCCs.^1^ The case is of a middle-aged man with numerous risk factors who underwent successful wide local excision of a scrotal basal cell carcinoma.

## 2 Case presentation

**Fig. 1:**
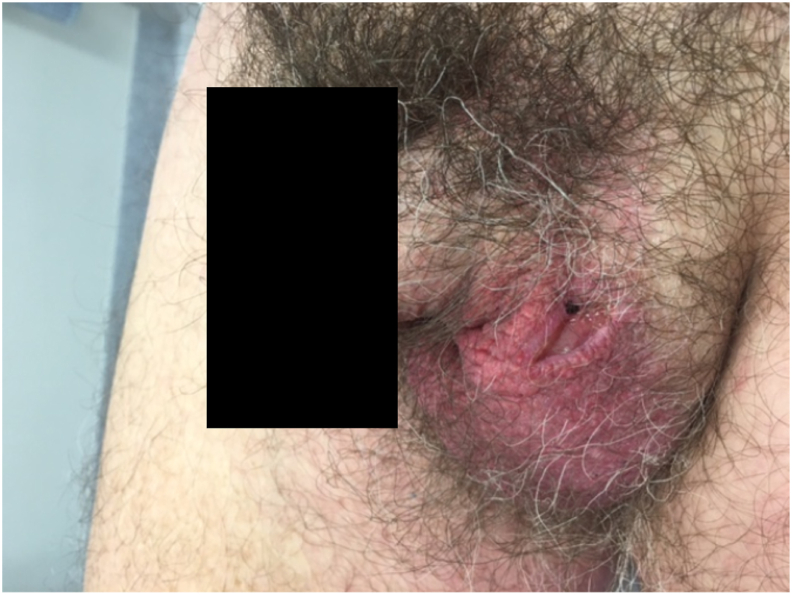
Pre-operative photo of the ulcerated lesion on the left anterior scrotum.

A 58-year-old Croatian male presented with an eight-month history of a gradually enlarging 19mm by 5mm lesion on the left anterior scrotum (Fig. 1). It was first noticed as a pimple-like lump, which eroded then ulcerated and did not heal. It was tender but never pruritic.

His medical background consisted of HPV-related genital warts, adrenal adenoma, 40-pack-year smoking history, ex-intravenous drug use, Hepatitis C now cleared, Hepatitis B and psoriasis. He was diagnosed with psoriasis at age 17 which had been persistent and widespread but without scrotal involvement. He was treated with phototherapy, methotrexate, DMARDS and biologics, with stable disease on Adalimumab (TNFa inhibitor) and then Secukinumab. He had two previous basal cell carcinomas on his back excised. There were no other skin cancers on less sun-exposed regions.

**Fig. 2:**
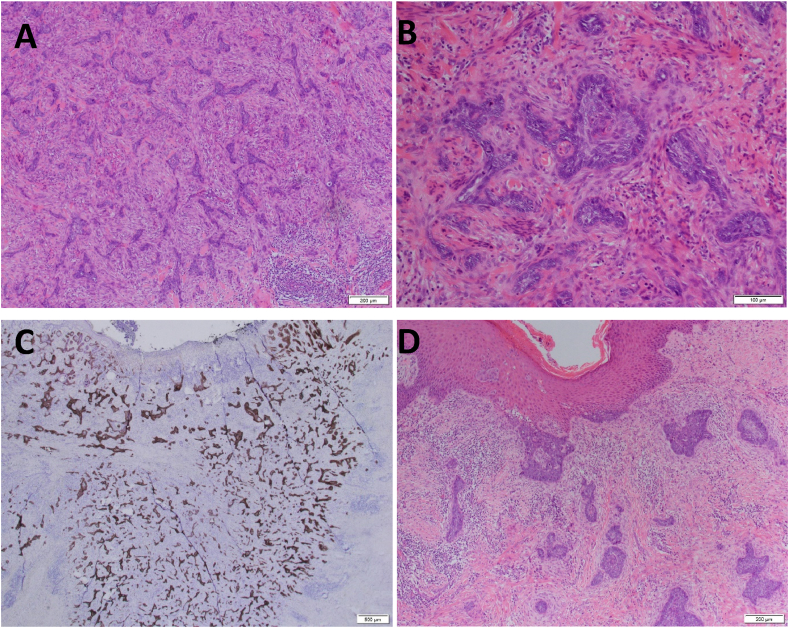
Histopathology of basal cell carcinoma showing infiltrative growth. Angulated nests of basaloid cells with peripheral palisading of tumour cells are seen. Immunochemically stained BerEP4 positive, but p16 negative.

Punch biopsy of the scrotal lesion was performed and showed an epithelial malignancy which favoured BCC. The patient underwent a wide local excision, superficial to tunica albuginea. Histopathology confirmed BCC with infiltrative growth (Fig. 2A). It was composed of angulated nests of basaloid cells with palisading of tumour cells at the periphery, and focal keratin pearls (Fig. 2B). It was immunochemically BerEP4 positive, but p16 negative (Fig. 2C). Although highly uncommon in non-sun-exposed sites, the tumour showed sufficient irregular growth and epidermal attachment to delineate it from a trichoepithelioma (Fig. 2D). The growth pattern was irregular and infiltrating, dissecting dermal collagen, and pushing between bundles of dartos muscle (depth 5.5mm). The surface was deeply ulcerated, and focal epidermal attachment were seen. The sample depth was 15mm, surgical margin was clear (closest point 1mm), with no perineural or lymphovascular invasion. Aligning with history, no psoriasiform change was seen.

There were no complications other than patient reported tissue tightening and slow healing due to immunosuppressive therapy. He was discharged at one year follow-up with private urologist and continues six monthly reviews with his rheumatologist.

## 3 Discussion

### 3.1 Diagnostic approach

Differentials for a solitary scrotal skin lesion should include BCC, squamous cell carcinoma, melanoma, extramammary Paget's disease, or pyoderma gangrenosum. Multiple lesions generally favour epidermal cysts, calcinosis, atypical infection (mycobacterial, fungal) or sexually transmitted infection (chancroid, syphilis). BCC may present as solitary lesion of varying appearance, including plaques, nodules and non-healing ulcers.^1^ Diagnosis can be made by clinical examination and dermoscopy. Definitive diagnosis is with excision and histopathology.

### 3.2 Risk factors

When BCC develops in a non-ultraviolet exposed site, it prompts consideration of other predisposing factors. These include decreased immunosurveillance, PTCH1, TP53 and other tumor-related genes, or it can be part of syndromes such as Basal cell nevus syndrome.^1^ Patients with psoriasis on TNF-inhibitors for more than 12 months have been shown to have an increased risk.^2^ Our patient had multiple risk factors for carcinogenesis including immune-modulating medication, chronic skin irritation, and smoking.

### 3.3 Management approach

Surgical, cryotherapy, topical pharmacological and photodynamic therapies are possible treatments for BCC.^3^ Wide local excision has the lowest 5-year recurrence rates.^3^ Scrotal rugosity complicates delineation of tumour margins, however tissue preservation prevents cosmetic defect.^4^ Imiquimod cream may be an adjunct to reduce BCC size and hence surgical defect.^3^ There would be a role for lymph node dissection and adjuvant chemotherapy or radiotherapy if metastasis was evident.^1^

### 3.4 Prognosis

In contrast to non-scrotal BCCs which have very low rate of metastasis (less than 0.5% after 11 years^5^), scrotal BCCs are much more likely to metastasise, with up to 20% at 24 months post-excision.^1^ Inguinal lymph node, lung, bone and skin are the most common sites of spread.^1^ The lack of subcutaneous fat and high vascularity of the scrotum may contribute to the more aggressive course of scrotal BCC.^4^ Involvement of the dartos muscle may be a poor prognostic indicator on final pathology.^1^ Given its unique behaviour, scrotal BCC patients should be monitored with thorough examination, and imaging especially in high-risk patients with dartos involvement, positive surgical margins or are immunosuppressed.^1^

## 4 Conclusion

In summary, BCC is rare in non-sun-exposed regions. Scrotal BCC is more likely to metastasise. This case highlights an approach to scrotal skin lesions, the risk factors and diagnosis of BCC. Management should consist of wide local excision and recommended follow up clinically with history, examination and imaging in high-risk patients.

## Declaration of competing interest

None declared.
